# Exploring the deactivation mechanism of human *β*_2_ adrenergic receptor by accelerated molecular dynamic simulations

**DOI:** 10.3389/fmolb.2022.972463

**Published:** 2022-08-30

**Authors:** Jianzhong Chen, Jian Wang, Qingkai Zeng, Wei Wang, Haibo Sun, Benzheng Wei

**Affiliations:** ^1^ School of Science, Shandong Jiaotong University, Jinan, China; ^2^ Center for Medical Artificial Intelligence, Shandong University of Traditional Chinese Medicine, Qingdao, China

**Keywords:** *β*_2_ adrenergic receptor, G protein-coupled receptors, GaMD simulations, free energy landscapes, deactivation mechanism

## Abstract

The *β*_2_ adrenergic receptor (β_2_AR), one of important members of the G protein coupled receptors (GPCRs), has been suggested as an important target for cardiac and asthma drugs. Two replicas of Gaussian accelerated molecular dynamics (GaMD) simulations are performed to explore the deactivation mechanism of the active β_2_AR bound by three different substrates, including the agonist (P0G), antagonist (JTZ) and inverse agonist (JRZ). The simulation results indicate that the Gs protein is needed to stabilize the active state of the β_2_AR. Without the Gs protein, the receptor could transit from the active state toward the inactive state. During the transition process, helix TM6 moves toward TM3 and TM5 in geometric space and TM5 shrinks upwards. The intermediate state is captured during the transition process of the active β_2_AR toward the inactive one, moreover the changes in hydrophobic interaction networks between helixes TM3, TM5, and TM6 and the formation of a salt bridge between residues Arg^3.50^ and Glu^6.30^ drive the transition process. We expect that this finding can provide energetic basis and molecular mechanism for further understanding the function and target roles of the β_2_AR.

## Introduction

The GPCRs represent the largest protein family correlating with signal transduction across membranes (Rasmussen et al., 2011a) and are responsible for the most of cellular responses to hormones and neurotransmitters as well as the senses of sight, olfaction and taste (Isberg et al., 2015). The *β*_2_ adrenergic receptor (β_2_AR) is one of the best characterized model among the GPCRs because of its three-type identified ligands, namely agonist, antagonist and inverse agonist (Li et al., 2013). Similar to the other members of GPCRs, binding of an agonist leads to a transition of β_2_AR conformation toward the active state, which further induces association of β_2_AR with the G-proteins and various intracellular responses (Trincavelli et al., 2010). Differently, binding of an inverse agonist causes a shift of the receptor toward the inactive state, leading to unfavorable binding with the G-proteins thus blocking the signal transduction (Li et al., 2013). The presence of an antagonist blocks substrate binding site and in turn affects the activity of β_2_AR. The previous studies verified the active or inactive states of β_2_AR consist of various receptor conformations with different signaling implications (Galandrin and Bouvier 2006; Kobilka and Deupi 2007). Hence it is critical to probe dynamics behavior of conformational changes induced by ligand bindings for understanding functions of β_2_AR and its target roles in drug design.

Based on significance of β_2_AR’s dynamics information in drug design toward treatment of ischemic, coronary artery, and inflammatory diseases, many experimental and theoretical works have focused on insights into conformational changes of β_2_AR (Miao and McCammon 2018; Liu et al., 2019a; Ishchenko et al., 2019). The crystal structure of the β_2_AR-Gs complex determined by Rasmussen et al. (2011b) suggest that the largest conformational change of β_2_AR involves an outward motion of 14 Å at the cytoplasmic end of transmembrane segment 6, termed as TM6. Human β_2_ARs bound by inverse agonists and antagonist exhibit that binding pocket of β_2_AR can accept compounds of various chemical and pharmacological properties with only minor local conformational changes (Wacker et al., 2010). The crystal structures of the ligand-bound β_1_AR and β_2_AR from Xu et al. (2021) uncover that the catecholamine binding pockets are identical between β_1_AR and β_2_AR, but the extracellular vestibules have different shapes and electrostatic properties. Liu et al. (2020) solve the structure of the AS408-bound β_2_AR and their study reveals molecular mechanism of AS408 stabilizing the inactive conformation of the β_2_AR. The structures of the β_2_AR adrenergic receptor bound by an orthosteric agonist and a compound 6FA from the work of Liu et al. (2019b) unveil mechanism of the β_2_AR regulation by an intracellular positive allosteric modulator. These experimental works provide significant structural basis for further investigating the function of the β_2_AR.

Apart from experimental studies, theoretical computations are also applied to probe the function and target roles of the β_2_AR. Supervised machine learning and dynamic network analysis from Chen et al. (2021c) show that binding of nanobody produces an allosteric effect on ligand-specific active states and triggers tighter and stronger local communication networks between the Nb80 and the ligand-associated sites. Microsecond-timescale molecular dynamics (MD) simulations on β_2_ARs in multiple wild-type and mutant states indicate that conformations of inactive β_2_AR reach an equilibrium between the lock formed state and the lock broken one, whether or not the cocrystallized ligand is present (Dror et al., 2009). Recently, experimental and theoretical insights into conformational changes of β_2_AR induced by ligand bindings are ongoing (Li et al., 2013; Staus et al., 2016; Liu et al., 2019a; Stanek et al., 2019; Wang and Miao 2019). Despite great success obtained in the previous works, dynamic information relating with the state changes between the active and inactive β_2_AR is still insufficient. Therefore, it is highly essential to further probe conformational changes of the β_2_AR for understanding the activity regulation and function of the β_2_AR.

Conventional molecular dynamics (cMD) simulations (Xue et al., 2018a; Xue et al., 2018b; Sun et al., 2021a) and predictions of binding free energies (Sun et al., 2014; Sun et al., 2021b) can provide useful dynamic information and energetic basis for elucidating functions of targets. Recently, a more efficient sampling technology, Gaussian accelerated molecular dynamics (GaMD) simulation (Miao et al., 2015; Wang et al., 2021), is proposed by Miao and McCammon (2016) to improve conformational sampling of targets, moreover this technology has obtained great successes in exploration of ligand-induced conformational alterations of targets (Wang and Miao 2020; Chen et al., 2021a; Chen et al. 2021b; Chen et al. 2022). More importantly, Draper-Joyce et al. (2021) use GaMD and cMD simulations to probe positive allosteric mechanisms of adenosine A1 receptor-mediated analgesia and their results verify that GaMD simulations better capture conformation transitions between the active and inactive states of adenosine A1 receptor than cMD simulations. In this work, three different ligands, namely agonist P0G (Rasmussen et al., 2011a), antagonist JTZ (Wacker et al., 2010) and (Wacker et al., 2010) are selected to probe deactivation mechanism of the β_2_AR and decipher free energy profiles that affect conformational transition of the β_2_AR. The structures of P0G, JTZ and JRZ are displayed in supporting information (Supplementary Figure S1). To obtain rational conformational samplings, Two replicas of GaMD simulations are performed on the P0G-bound active β_2_AR with the Gs protein (Rasmussen et al., 2011b), P0G-, JTZ- and JRZ-bound active β_2_AR without the Gs protein. Free energy landscapes (FELs) are built by using reaction coordinates from the GaMD trajectories to reveal energetic basis and clarify dynamics information underlying the deactivation process of the β_2_AR. This work is expected to provide molecular mechanism for deeply understanding the function of the β_2_AR.

## Methods

### System preparation

The initial coordinates of the P0G-bound active β_2_AR with the Gs protein used for GaMD simulations are taken from protein data bank (PDB) and its entry is 3SN6 (Rasmussen et al., 2011a). The active β_2_AR removing the Gs protein from 3SN6 is superimposed with the crystal structure 3P0G (Rasmussen et al., 2011b), 3NYA (Wacker et al., 2010) and 3NY8 (Wacker et al., 2010) to ,respectively generate the P0G-, JTZ-, and JRZ-bound active β_2_AR without the Gs protein. Among four complexes, the active β_2_AR and the Gs protein together with ligands P0G, JRZ and JTZ are retained at the starting model. All missing hydrogen atoms are bonded to their corresponding heavy atoms by using the Leap module in Amber 20 (Salomon-Ferrer et al., 2013a). The protonation states of residues in β_2_AR are checked by using the program PROPKA (Li et al., 2005; Bas et al., 2008) and the rational protonation states are assign to the β_2_AR. The active β_2_AR is inserted into a palmitoyl-oleoylphosphatidyl-choline (POPC) bilayer with all overlapping lipid molecules removed. The above mentioned complexes are solved at the water box consisting of TIP3P water model (Jorgensen et al., 1983). The amber force field *ff*19SB (Tian et al., 2020) and amber lipid force field LIPID 14 (Dickson et al., 2014) are used to respectively produce force filed parameters of the Gs protein and β_2_AR as well as lipid POPC. The atomic partial charges in general AMBER force field (GAFF) and the second generation of GAFF (GAFF2) can generate reliable force field parameters of small molecules and it is used to accurately predict the affinity and binding selectivity of ligands to receptors (Wang et al., 2004; He et al., 2020). Thus, GAFF2 is adopted to yield the force field parameters of ligands P0G, JTZ, and JRZ. The method of the Austin Model 1 with bond charge correction (AM1-BCC) (Jakalian et al., 2000; Jakalian et al., 2002) method is applied to produce the atomic charges of P0G, JTZ, and JRZ through the Antechamber tool in Amber (Wang et al., 2006). The system charges are then neutralized at salt environment of 0.15 M NaCl. All of the aforementioned operations are finished by using the web-sever CHARMM-GUI (Wu et al., 2014; Lee et al., 2019).

### GaMD simulations

To remove high-energy contacts between atoms of simulated systems, each system is optimized using the steepest descent minimization of 50,000 steps and the conjugate gradient one of another 50,000 steps. Subsequently, the systems endure a 2-ns soft heating process from 0 to 310 K by restraining heavy atoms of the ligand-bound active β_2_AR with or without the Gs proteins with 1 kcal/(mol Å^2^) harmonic constant in a constant number, volume and temperature (NVT) ensemble. Then, four systems are further equilibrated for 2 ns in a constant number, pressure and temperature (NPT) ensemble at 1 atm and 310 K by using the same restraints as in the previous NVT simulation. After that, a 3-ns cMD simulation is performed to examine potential energy statistics, involving the maximum, minimum, average, and standard deviation of four systems. Then, a 30-ns GaMD equilibration with the boost potential is run on the P0G-, JTZ-, and JRZ-bound active β_2_AR without the Gs proteins, while a 40-ns GaMD equilibration is done on that with the Gs proteins. Finally, two separate 1-μs GaMD simulations are conducted with randomized initial atomic velocities to relax each system. During all current cMD and GaMD simulations, chemical bonds between hydrogen atoms and heavy ones are restrained with the SHAKE algorithm (Ryckaert et al., 1977). The temperatures of the systems are regulated with the Langevin dynamics with a collision frequency of 2.0 ps^−1^ (Izaguirre et al., 2001). An appropriate cutoff value of 12 Å is adopted to perform calculations of electrostatic interactions with the particle mesh Ewald method (Essmann et al., 1995) and estimations of van der Waals interactions. To be convenient for the post processing analysis, GaMD trajectories of two separate replicas are combined into a single joined trajectory (SJT). The PyReweighting toolkit (Miao et al., 2014) is employed to reweight the data stemming from the CPPTRAJ analysis on the SJT (Roe and Cheatham 2013) and recover the original free energy of four simulated systems. The details of GaMD simulations and principal component analysis (PCA) have been clarified in our previous works (Chen et al., 2021d). All simulations through this current study are run by mean of the program pmemd.cuda inlayed in Amber 20 (Salomon-Ferrer et al., 2013b).

## Results

### Structural stability of the P0G-bound β_2_AR with the Gs protein

**FIGURE 1:**
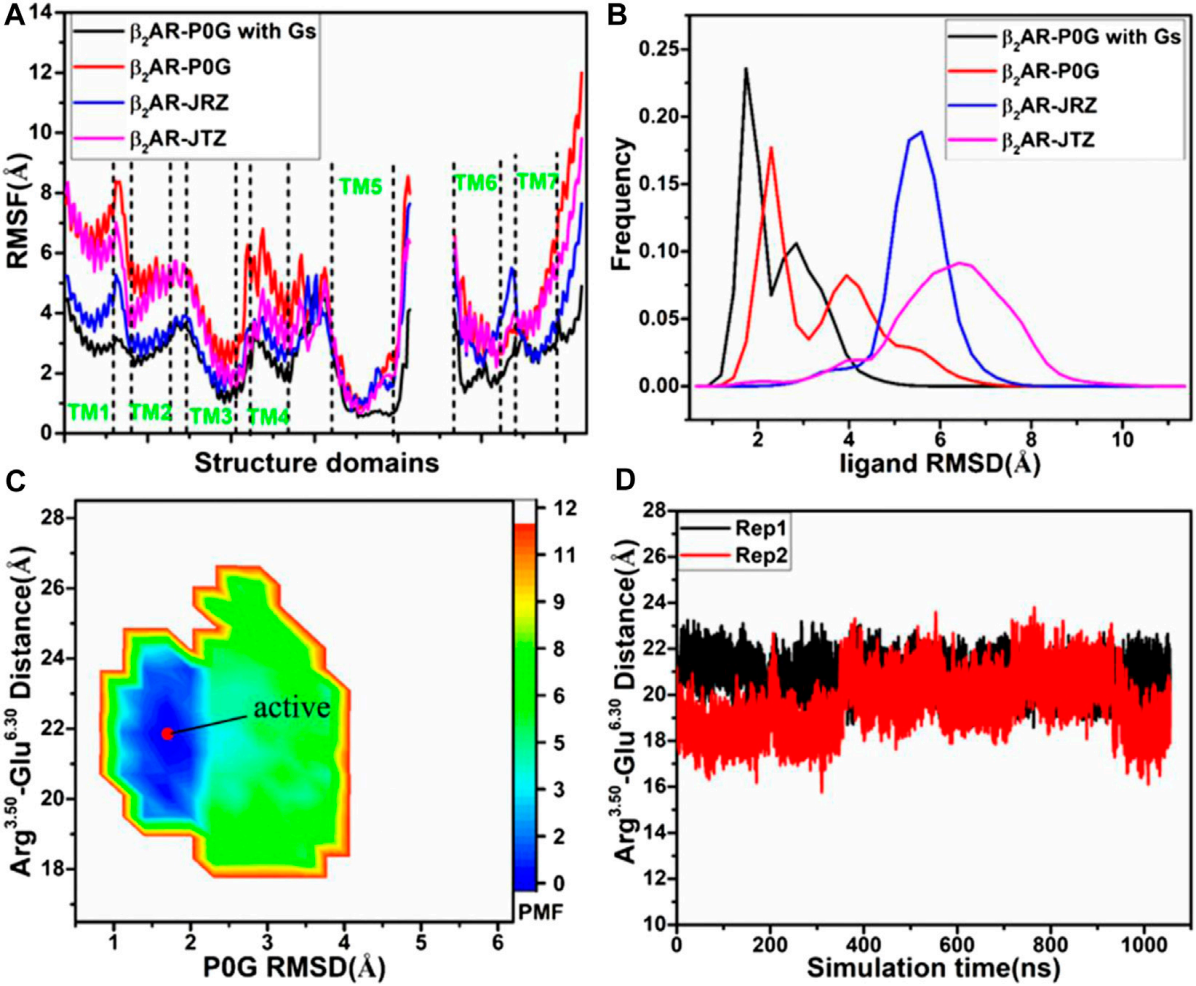
Structural fluctuation and free energy information: **(A)** RMSFs of the C_α_ atoms from β_2_AR in four simulated systems, **(B)** RMSDs of non-hydrogen atoms from P0G, JRZ, and JTZ in four simulated systems, **(C)** free energy landscape of the P0G-bound β_2_AR with the Gs protein constructed using the distances between the C_α_ atoms of residues Arg^3.50^ and Glu^6.30^ and the RMSD of P0G, and **(D)** the evolution of the distance between the C_α_ atoms of residues Arg^3.50^ and Glu^6.30^ as the simulated time. PMF is scaled in kcal/mol.

To understand structural flexibility of the β_2_AR in the binding environment, root-mean-square fluctuations (RMSFs) of the C_α_ atoms from four simulated systems are computed by averaging on two replicas (Figure 1A). Compared to the P0G-bound β_2_AR with the Gs protein, structural flexibility of all domains in the P0G-, JRZ-, and JTZ-bound β_2_AR without the Gs protein are strengthened, verifying that the Gs protein is needed to stabilize the active state of the β_2_AR. Except for the loops linking helixes, structural flexibility of helixes TM1, TM2, TM5, and TM6 from the P0G-, JRZ-, and JTZ-bound β_2_AR without the Gs protein are enhanced relative to that with the Gs protein. Meanwhile, structural flexibility of TM2 and TM4 from the P0G- and JTZ-bound β_2_AR without the Gs protein is also increased compared to that with the Gs protein. To access the effect of the Gs protein on global flexibility of the β_2_AR, molecular surface areas (MSAs) of the β_2_AR are calculated by using the atomic coordinates (Supplementary Figure S2). It is observed that the MSAs of the P0G-, JRZ-, and JTZ-bound β_2_AR without the Gs protein are increased by ∼702, 1,032, and 684 Å^2^ relative to the P0G-bound β_2_AR with the Gs protein, respectively, suggesting that binding of the Gs protein decreases global flexibility of the β_2_AR.

To unveil structural fluctuations of ligands P0G, JRZ, and JTZ in binding pocket of the β_2_AR, root-mean-square deviations (RMSDs) of non-hydrogen atoms from these three ligands are calculated and their frequency distribution are displayed at Figure 1B. Similar fluctuation tendency is observed in the RMSD plot of P0G in the β_2_AR with/without the Gs protein, which includes two peak values although larger RMSD values are identified in the simulations of system without the Gs protein. Meanwhile, the RMSDs of JRZ and JTZ in the β_2_AR without the Gs protein are larger than that in the P0G-bound β_2_AR with the Gs protein. The aforementioned results indicate that the structural fluctuations of P0G, JRZ, and JTZ in the binding pocket are strengthened without binding of the Gs protein, suggesting there are allosteric effects between the agonist and the G protein binding pockets. These results are consistent with the result that the G protein binding can increases the binding affinity of agonist (Rasmussen et al., 2011a).

To reveal the effect of an agonist binding on free energy profile of the active β_2_AR with the Gs protein, the distance of the C_α_ atom in Arg^3.50^ away from that in Glu^6.30^ and the RMSD of non-hydrogen atoms from P0G are used as reaction coordinates to build the FEL. The distance between the C_α_ atoms of Arg^3.50^ and Glu^6.30^ can reflect conformational transition between the active state and inactive one of the β_2_AR, while the RMSD of P0G can exhibit its structural fluctuation in the β_2_AR. The constructed FEL is displayed in Figure 1C. For the P0G-bound active β_2_AR with the Gs protein, GaMD simulations only capture a low energetic state (Figure 1C) with a RMSD of 0.92 Å relative to the crystal structure 3SN6, agreeing with the X-ray structure (Supplementary Figure S3). The evolution of the distance between the C_α_ atoms of Arg^3.50^ and Glu^6.30^ as the simulation time is calculated (Figure 1D) and the information shows that the β_2_AR always keeps the active state due to binding of the β_2_AR, further verifying that the Gs protein is needed for stabilizing the active state of the β_2_AR.

### Free energy landscapes of P0G, JRZ, and JTZ-bound active β_2_AR without the Gs protein

**FIGURE 2:**
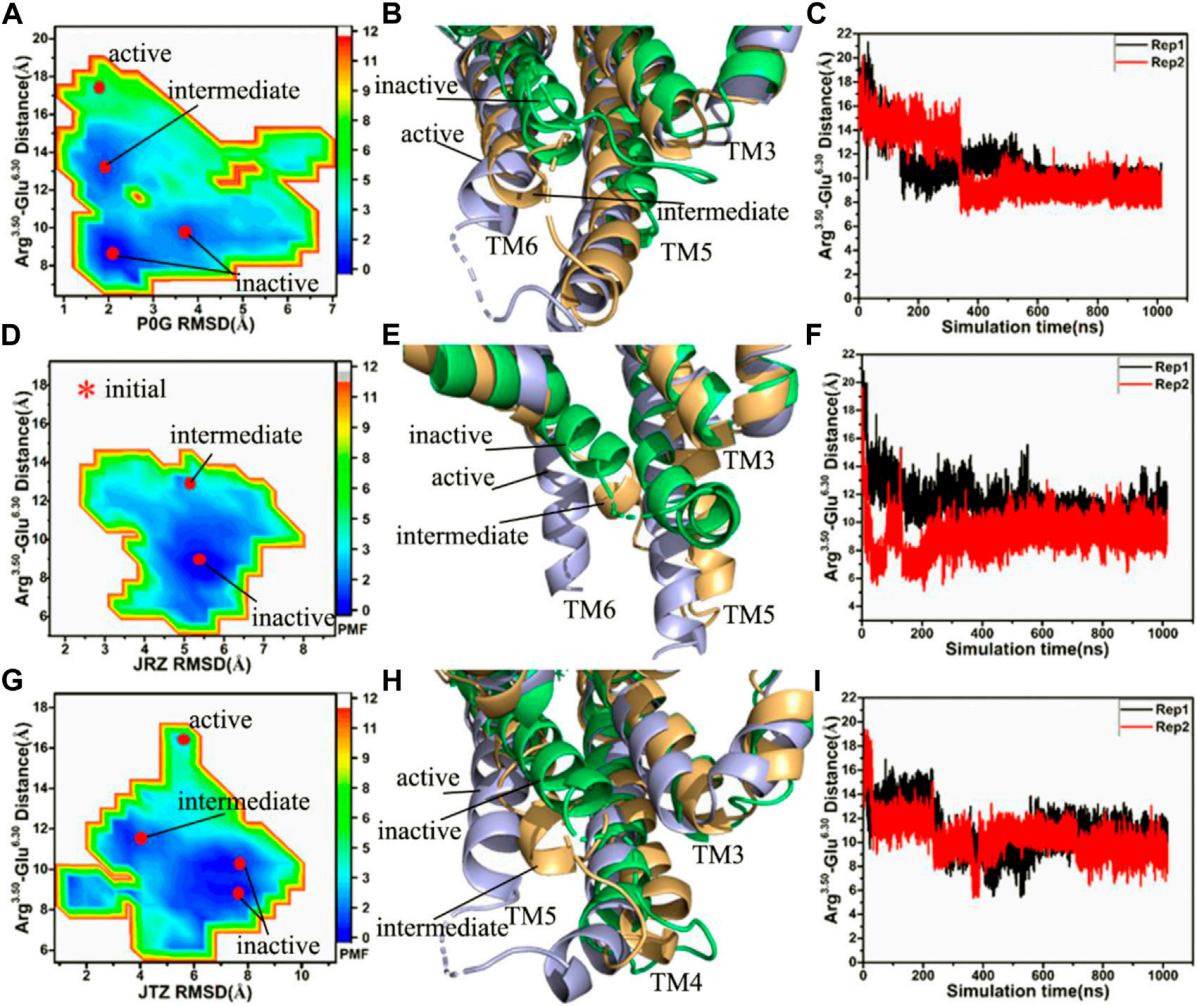
Free energy landscapes and conformational changes: **(A,D,G)** corresponding to free energy landscapes of the P0G-, JRZ-, and JTZ-bound β_2_AR without the Gs protein constructed using the distances between the C_α_ atoms of residues Arg^3.50^ and Glu^6.30^ and the RMSDs of ligands, respectively, **(B,E,H)** representing structural superimposition of the P0G-, JRZ, and JTZ-bound active, intermediate and inactive β_2_AR without binding of the Gs protein, individually, and **(C,F,I)** indicating the evolution of the distance between the C_α_ atoms of residues Arg^3.50^ and Glu^6.30^ as the simulated time in the P0G-, JRZ-, and JTZ-bound β_2_AR without the Gs protein, separately. PMF is scaled in kcal/mol.

To investigate the effect of binding of P0G, JRZ, and JTZ on the activity of the β_2_AR, the same reaction coordinates as that used in the P0G-bound β_2_AR with the Gs protein are utilized to construct the FELs of the P0G-, JRZ-, and JTZ-bound active β_2_AR without the Gs protein (Figure 2). The time courses of the distance between the C_α_ atoms of Arg^3.50^ and Glu^6.30^ and structural superimposition of the β_2_AR from different energetic states are also shown in Figure 2.

Without the Gs protein, four low energetic states are identified in the P0G-bound receptor simulations, including an active state, an intermediate state and two inactive states (Figure 2A), suggesting that GaMD simulations have successfully captured the deactivation process of the active β_2_AR. The structural superimposition of the active, intermediate and inactive states reveals the transition pathway of the active β_2_AR toward the inactive one (Figure 2B; Supplementary Figure S4). Two interesting phenomena are observed: 1) the bottom of helix TM6 goes away from the position of the active β_2_AR and is close to the helixes TM3 and TM5, 2) the bottom of helix TM5 shrink upwards, especially for the inactive sate (Figure 2B; Supplementary Figure S4). The time course of the distance between the C_α_ atoms of Arg^3.50^ and Glu^6.30^ in two separate replicas is provided in Figure 2C, indicating that GaMD simulations capture the transition of the active β_2_AR into the inactive state. The time evolution of the distance of the nitrogen atom (N) in Arg^3.50^ away from the oxygen atom (O) in Glu^6.30^ in two separate replicas is depicted in Supplementary Figure S5 and the average value of this distance in two simulations Rep1 and Rep2 are ∼5.1 and 10.8 Å, respectively, indicating that a strong salt bridge interaction is formed between residues Arg^3.50^ and Glu^6.50^ during the simulation Rep1. The above presented conformational changes of helixes TM5 and TM6 are also observed at the work of Rasmussen et al. (2011b).

In the JRZ-bound active β_2_AR without the Gs protein, two low energetic states, namely an intermediate state and an inactive one, appear at the FEL (Figure 2D). To clarify the transition process, the intermediate and inactive structures detected by GaMD simulations are superimposed with the initial optimized structure (initial) of the JRZ-bound active β_2_AR without the Gs protein (Figure 2E; Supplementary Figure S6). The superimposed results show similar phenomena to the P0G-bound active β_2_AR without the Gs protein: 1) the bottom of helix TM6 leaves the position of the active β_2_AR and is close to two helixes TM3 and TM5, 2) the bottom of helix TM5 shrinks upwards, which verifies that the transition process of the active β_2_AR toward the inactive state exists in our current GaMD simulations. To further confirm this transition process, the equilibrated phases in two separate replicas are also used to compute the evolution of the distance between the C_α_ atoms of Arg^3.50^ and Glu^6.30^ as the simulation time and the results are depicted in Figure 2F and Supplementary Figures S7A,B. It is found that the JRZ-bound active β_2_AR without the Gs protein quickly realizes the transition of the active β_2_AR toward the inactive state before the first 20 ns of the equilibration in two separate simulations (Supplementary Figure S7B). Meanwhile, time evolution of the distance of the nitrogen atom (N) in Arg^3.50^ away from the oxygen atom (O) in Glu^6.30^ in two separate replicas are also estimated (Supplementary Figure S7C) and the average values of this distance in two separate simulations Rep1 and Rep2 are ∼5.1 and 12.0 Å, respectively, indicating that a stable salt bridge interaction is formed through the simulation Rep1.

In the JTZ-bound active β_2_AR without the Gs protein, GaMD simulations detect four different energetic states, including an active state, an intermediate state and two inactive states, which captures a transition process of the active β_2_AR toward the inactive state (Figure 2G). The alignment of the β_2_AR located at the active, intermediate and inactive states display a transition pathway of the active β_2_AR toward the inactive one (Figure 2H; Supplementary Figure S8). Meanwhile, this structural alignment also reveals two interesting results: 1) the bottom of helix TM6 leaves the active position of the β_2_AR and moves toward helixes TM3 and TM5, 2) the bottom of TM5 generates an upward shrinkage. The function of the distance between the C_α_ atoms of Arg^3.50^ and Glu^6.30^ as the simulation time is computed with the SJT (Figure 2I) and the information suggests that both the simulations Rep1 and Rep2 capture the transformation of the active β_2_AR into the inactive state. The time evolution of the distance between the nitrogen atom (N) in Arg^3.50^ and the oxygen atom (O) in Glu^6.30^ is exhibited at Supplementary Figure S9. The average values of the Arg^3.50^-Glu^6.30^ NO distance in two separate simulations Rep1 and Rep2 are 5.1 and 5.0 Å, respectively, indicating that a stable salt bridge interaction between Arg^3.50^ and Glu^6.30^ is produced during the simulations Rep1 and Rep2. The study of Dror et al. (2009) also captures the transition process of the active β_2_AR toward the inactive state and the formation of the salt bridge between Arg^3.50^ and Glu^6.30^, which supports our current results.

As shown in Figures 2B,E,H, bindings of P0G, JRZ, and JTZ all induce the transition of the active β_2_AR without the Gs protein toward the inactive state. However, the transition process endures different time in the P0G-, JRZ-, and JTZ-bound β_2_AR without the Gs protein. For the P0G-bound β_2_AR without the Gs protein, the Rep1 and Rep2 simulations, respectively spend 152 and 339 ns in realizing the transition process and their averaged time is 245.5 ns. As for the JRZ-bound β_2_AR, the Rep1 and Rep2 simulations individually take 12 and 9 ns to capture the transition process and their average time is 10.5 ns. In the case of the JTZ-bound β_2_AR without the Gs protein, the Rep1 and Rep2 simulations separately spend 225 and 48 ns in achieving the transition process and their averaged time is 136.5 ns. Thus, the agonist P0G and the inverse agonist JRZ, respectively take the longest time and the shortest time to realize the transformation of the active β_2_AR into the inactive one, which agrees with the biology process of three ligands (Michel et al., 2020).

To clarify main forces driving the deactivation process of the active β_2_AR, the interactions of key residues from helixes TM3, TM5, and TM6 in the active, intermediate and inactive states of the β_2_AR are analyzed (Supplementary Figure S10). In the active β_2_AR, a cation-π interaction appears between residues Arg^3.50^ and Tyr^7.53^, meanwhile residues Ile^6.78^ and Leu^6.37^, respectively produce the CH-π interactions with residues Tyr^5.58^ and Phe^5.62^ (Supplementary Figure S10A). In the P0G-bound intermediate state of the β_2_AR, the CH-π interaction networks of Met^5.54^ with Phe^6.44^, Met^6.41^ and Leu^6.37^ with Tyr^5.58^ and Leu^6.34^ with Phe^5.62^ are detected, separately (Supplementary Figure S10B), while in the P0G-bound inactive state of the β_2_AR, the CH-π interaction of Leu^3.43^ with Phe^6.44^ and Met^6.41^ with Tyr^5.58^ together the CH-CH interactions between Val^5.61^ and Leu^6.34^ are identified (Supplementary Figure S10C). The CH-π interactions of Leu^5.51^ and Met^5.54^ with Phe^6.44^, Leu^6.37^ with Tyr^5.58^ and Phe^5.62^ and Leu^6.34^ with Phe^5.62^ appear at the JRZ-bound intermediate state of the β_2_AR (Supplementary Figure S10D), but that of Leu^3.43^ with Phe^6.44^, Leu^6.49^ with Phe^5.47^, Met^6.41^ with Tyr^5.58^ and Leu^6.34^ with Phe^5.62^ are recognized at the JRZ-bound inactive state of the β_2_AR (Supplementary Figure S10E). Except for the π-π interaction between His^6.31^ and Phe^5.62^ from the JTZ-bound inactive β_2_AR, the CH-π interaction networks in the JTZ-bound intermediate and inactive states of the β_2_AR are similar to that in the JRZ-bound intermediate and inactive states of the β_2_AR (Supplementary Figures S10F,G). By comparison with the active β_2_AR, the hydrophobic interaction networks in the substrate-bound intermediate and inactive states of the β_2_AR change, and finally two common interactions between TM3 and TM6, including the CH-π interaction of Leu^3.34^ with Phe^6.44^ and a salt bridge between residues Arg^3.50^ and Glu^6.30^, are formed in the inactive state of the β_2_AR, which stabilizes the inactive state of the β_2_AR, thus the changes in hydrophobic interaction networks among TM3, TM5 and TM6 drive the deactivation process of the active β_2_AR.

### Concerted motions of the β_2_AR revealed by principal component analysis

**FIGURE 3:**
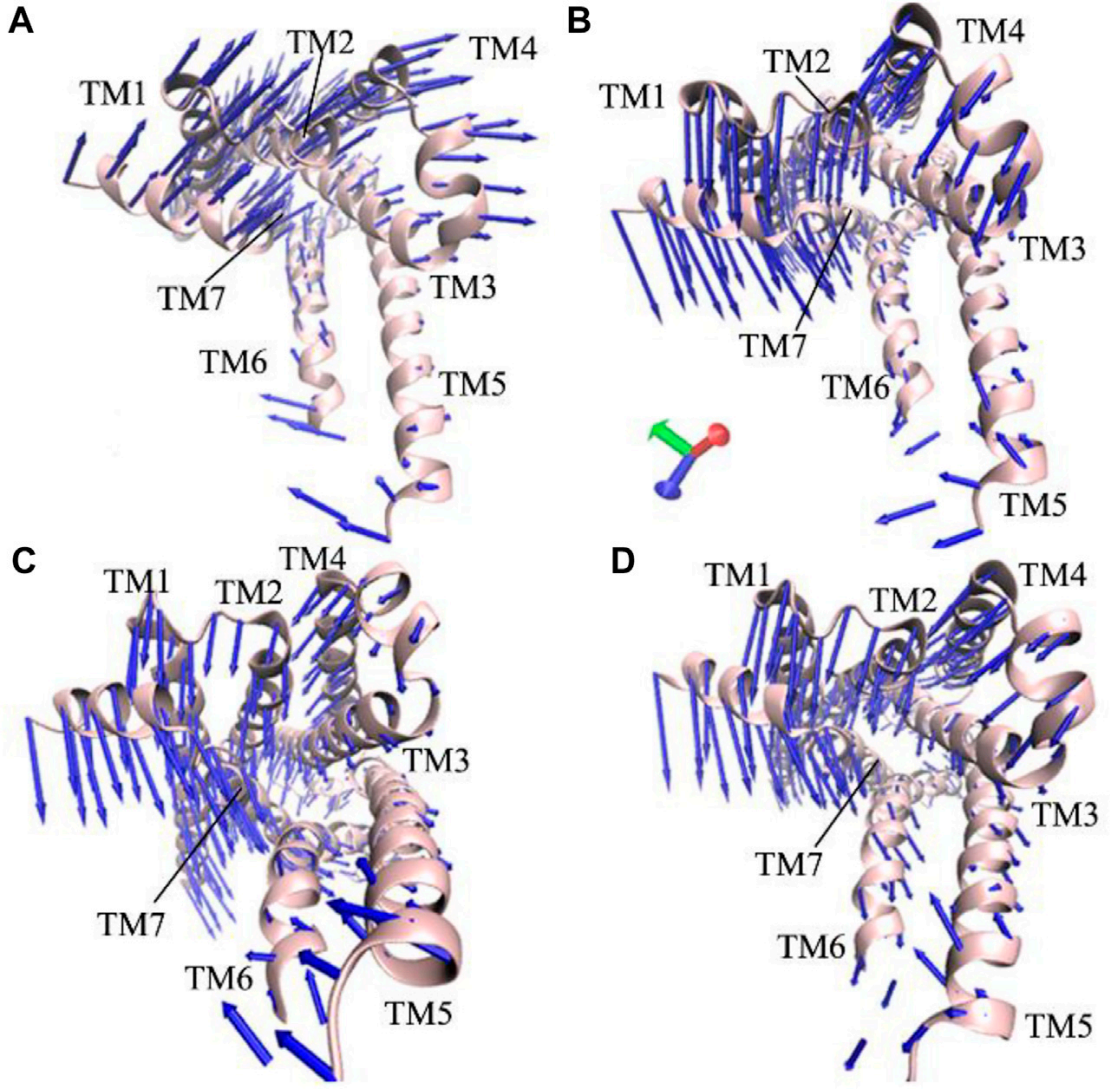
Concerted motions of structural domains from the β_2_AR: **(A)** the P0G-bound active β_2_AR with the Gs protein, and **(B–D)** corresponding to the P0G-, JTZ-, and JRZ-bound active β_2_AR without the Gs protein, respectively.

The previous FEL analyses reveal that GaMD simulations capture the transition of the active β_2_AR toward the inactive one, which certainly generates vital effect on dynamics behavior of the β_2_AR. To check this issue, PCA is performed on the four simulated systems. The first eigenvector arising from PCA is visualized and the results are plotted at Figure 3. It is observed that structure domains in the β_2_AR exhibit highly concerted motions and binding of the Gs protein affects dynamics behavior of the β_2_AR. The helixes TM1-TM4 and the loops at the top of the β_2_AR have an upper right motion and they tend to leave the helixes TM5 and TM6 in the P0G-bound active β_2_AR with the Gs protein (Figure 3A). However, by comparison with the P0G-bound β_2_AR with the Gs protein, the helixes TM1-TM4 and the loops at the top of the β_2_AR have a high concerted motion toward the helixes TM5 and TM6 in the P0G-, JTZ-, and JRZ-bound β_2_AR without the Gs protein, moreover the helixes TM5 and TM6 have a tendency being close to each other (Figures 3B–D). These different dynamics behavior of structural domains from the β_2_AR in the states with or without the Gs protein may form a main force to drive the transition of the active β_2_AR bound by P0G, JRZ, and JTZ toward the inactive state.

## Discussion

Agonists, antagonists and inverse agonists play significant roles in regulation on the activity of the β_2_AR. Insights into deactivation mechanism of the active β_2_AR are of high significance for understanding the function and target roles of the β_2_AR. To capture the transition process from the active β_2_AR to the inactive state, we construct four simulated systems by using the active β_2_AR, including the P0G-bound active β_2_AR with the Gs protein and the P0G-, JTZ-, and JRZ-bound active β_2_AR without the Gs protein. 2-μs GaMD simulations are performed to sample conformational space of the β_2_AR in different binding environment, in which the transition process from the active β_2_AR to the inactive state is successfully captured.

FELs are constructed by using the distances of the C_α_ atom in Arg^3.50^ away from that in Glu^6.30^ and the RMSDs of three ligands as reaction coordinates to reveal the changes in different energetic states of the β_2_AR. Our current GaMD simulations identify more low energetic states in the P0G-, JTZ-, and JRZ-bound active β_2_AR without the Gs protein than in the P0G-bound active β_2_AR with the Gs protein, indicating that binding of the Gs protein can stabilize the active state of the β_2_AR. Similar results are found in the RMSF analyses, which suggested that much lower flexibility is identified in the P0G-bound β_2_AR with Gs protein compared to the P0G-, JRZ-, and JTZ-bound systems without the Gs protein.

Both FELs and the time evolution of the distances between the C_α_ atoms of Arg^3.50^ and Glu^6.30^ suggest that GaMD simulations capture the transition process of the P0G-, JTZ, and JRZ-bound active β_2_AR toward the inactive state and identify the transition pathway. During the transition process, the helix TM6 leaves the position of the active β_2_AR and is close to the helixes TM3 and TM6, which is in consistence with the changes in concerted motions of the β_2_AR uncovered by PCA. The structures of the P0G-bound active, JRZ-bound inactive and JTZ-bound inactive β_2_AR without the Gs protein captured by GaMD simulations are respectively superimposed with their corresponding crystal structures 3P0G, 3NY8, and 3NYA and their RMSDs are 0.95, 1.34, and 0.94 Å, separately, indicating that our captured energetic structures are in good agreement with the experimental structures. More importantly, our GaMD simulations of the P0G-, JTZ-, and JRZ-bound active β_2_AR without the Gs protein capture the formation of a salt bridge interaction between residues Arg^3.50^ and Glu^6.30^ and the changes in hydrophobic interaction networks among TM3, TM5 and TM6. Thus long-range electrostatic interaction stemming from the salt bridge and the changes of hydrophobic interaction networks play an important role in the deactivation process. This study is expected to provide dynamics information for deeply understanding the function and target roles of the β_2_AR.

## Data Availability

The original contributions presented in the study are included in the article/Supplementary Material, further inquiries can be directed to the corresponding authors.

## References

[B1] Bas D. C. Rogers D. M. Jensen J. H. (2008). Very fast prediction and rationalization of pKa values for protein–ligand complexes. Proteins 73, 765–783. 10.1002/prot.22102 18498103

[B2] Chen J. Wang L. Wang W. Sun H. Pang L. Bao H. (2021d). Conformational transformation of switch domains in GDP/K-Ras induced by G13 mutants: An investigation through Gaussian accelerated molecular dynamics simulations and principal component analysis. Comput. Biol. Med. 135, 104639. 10.1016/j.compbiomed.2021.104639 34247129

[B3] Chen J. Zhang S. Wang W. Pang L. Zhang Q. Liu X. (2021a). Mutation-induced impacts on the switch transformations of the GDP- and GTP-bound K-ras: Insights from multiple replica Gaussian accelerated molecular dynamics and free energy analysis. J. Chem. Inf. Model. 61, 1954–1969. 10.1021/acs.jcim.0c01470 33739090

[B4] Chen J. Zhang S. Wang W. Sun H. Zhang Q. Liu X. (2021b). Binding of inhibitors to BACE1 affected by pH-dependent protonation: An exploration from multiple replica Gaussian accelerated molecular dynamics and MM-GBSA calculations. ACS Chem. Neurosci. 12, 2591–2607. 10.1021/acschemneuro.0c00813 34185514

[B5] Chen J. Zhang S. Zeng Q. Wang W. Zhang Q. Liu X. (2022). Free energy profiles relating with conformational transition of the switch domains induced by G12 mutations in GTP-bound KRAS. Front. Mol. Biosci. 9, 912518. 10.3389/fmolb.2022.912518 35586192 PMC9108337

[B6] Chen Y. Fleetwood O. Pérez-Conesa S. Delemotte L. (2021c). Allosteric effect of nanobody binding on ligand-specific active states of the β2 adrenergic receptor. J. Chem. Inf. Model. 61, 6024–6037. 10.1021/acs.jcim.1c00826 34780174 PMC8715506

[B7] Dickson C. J. Madej B. D. Skjevik Å. A. Betz R. M. Teigen K. Gould I. R. (2014). Lipid14: The amber lipid force field. J. Chem. Theory Comput. 10, 865–879. 10.1021/ct4010307 24803855 PMC3985482

[B8] Draper-Joyce C. J. Bhola R. Wang J. Bhattarai A. Nguyen A. T. N. Cowie-Kent I. (2021). Positive allosteric mechanisms of adenosine A1 receptor-mediated analgesia. Nature 597, 571–576. 10.1038/s41586-021-03897-2 34497422 PMC8711093

[B9] Dror R. O. Arlow D. H. Borhani D. W. Jensen M. Ø. Piana S. Shaw D. E. (2009). Identification of two distinct inactive conformations of the β_2_-adrenergic receptor reconciles structural and biochemical observations. Proc. Natl. Acad. Sci. U. S. A. 106, 4689–4694. 10.1073/pnas.0811065106 19258456 PMC2650503

[B10] Essmann U. Perera L. Berkowitz M. L. Darden T. Lee H. Pedersen L. G. (1995). A smooth particle mesh Ewald method. J. Chem. Phys. 103, 8577–8593. 10.1063/1.470117

[B11] Galandrin S. Bouvier M. (2006). Distinct signaling profiles of β_1_ and β_2_ adrenergic receptor ligands toward adenylyl cyclase and mitogen-activated protein kinase reveals the pluridimensionality of efficacy. Mol. Pharmacol. 70, 1575–1584. 10.1124/mol.106.026716 16901982

[B12] He X. Man V. H. Yang W. Lee T.-S. Wang J. (2020). A fast and high-quality charge model for the next generation general AMBER force field. J. Chem. Phys. 153, 114502. 10.1063/5.0019056 32962378 PMC7728379

[B13] Isberg V. Mordalski S. Munk C. Rataj K. Harpsøe K. Hauser A. S. (2015). GPCRdb: An information system for G protein-coupled receptors. Nucleic Acids Res. 44, D356–D364. 10.1093/nar/gkv1178 26582914 PMC4702843

[B14] Ishchenko A. Stauch B. Han G. W. Batyuk A. Shiriaeva A. Li C.(2019). Toward G protein-coupled receptor structure-based drug design using X-ray lasers. IUCrJ 6, 1106–1119. 10.1107/S2052252519013137 PMC6830214 31709066

[B15] Izaguirre J. A. Catarello D. P. Wozniak J. M. Skeel R. D. (2001). Langevin stabilization of molecular dynamics. J. Chem. Phys. 114, 2090–2098. 10.1063/1.1332996

[B16] Jakalian A. Bush B. L. Jack D. B. Bayly C. I. (2000). Fast, efficient generation of high-quality atomic charges. AM1-BCC model: I. Method. J. Comput. Chem. 21, 132–146. 10.1002/(SICI)1096-987X(20000130)21:2<132:AID-JCC5>3.0.CO;2-P 12395429

[B17] Jakalian A. Jack D. B. Bayly C. I. (2002). Fast, efficient generation of high-quality atomic charges. AM1-BCC model: II. Parameterization and validation. J. Comput. Chem. 23, 1623–1641. 10.1002/jcc.10128 12395429

[B18] Jorgensen W. L. Chandrasekhar J. Madura J. D. Impey R. W. Klein M. L. (1983). Comparison of simple potential functions for simulating liquid water. J. Chem. Phys. 79, 926–935. 10.1063/1.445869

[B19] Kobilka B. K. Deupi X. (2007). Conformational complexity of G-protein-coupled receptors. Trends Pharmacol. Sci. 28, 397–406. 10.1016/j.tips.2007.06.003 17629961

[B20] Lee J. Patel D. S. Ståhle J. Park S.-J. Kern N. R. Kim S. (2019). CHARMM-GUI membrane builder for complex biological membrane simulations with glycolipids and lipoglycans. J. Chem. Theory Comput. 15, 775–786. 10.1021/acs.jctc.8b01066 30525595

[B21] Li H. Robertson A. D. Jensen J. H. (2005). Very fast empirical prediction and rationalization of protein pKa values. Proteins 61, 704–721. 10.1002/prot.20660 16231289

[B22] Li J. Jonsson A. L. Beuming T. Shelley J. C. Voth G. A. (2013). Ligand-dependent activation and deactivation of the human adenosine A2A receptor. J. Am. Chem. Soc. 135, 8749–8759. 10.1021/ja404391q 23678995 PMC4120839

[B23] Liu X. Kaindl J. Korczynska M. Stößel A. Dengler D. Stanek M. (2020). An allosteric modulator binds to a conformational hub in the β2 adrenergic receptor. Nat. Chem. Biol. 16, 749–755. 10.1038/s41589-020-0549-2 32483378 PMC7816728

[B24] Liu X. Masoudi A. Kahsai A. W. Huang L.-Y. Pani B. Staus D. P.(2019a). Mechanism of β_2_AR regulation by an intracellular positive allosteric modulator. Science 364, 1283–1287. 10.1126/science.aaw8981 31249059 PMC6705129

[B25] Liu X. Xu X. Hilger D. Aschauer P. Tiemann J. K. S. Du Y. (2019b). Structural insights into the process of GPCR-G protein complex formation. Cell. 177, 1243–1251.e1212. 10.1016/j.cell.2019.04.021 31080070 PMC6991123

[B26] Miao Y. Feher V. A. McCammon J. A. (2015). Gaussian accelerated molecular dynamics: Unconstrained enhanced sampling and free energy calculation. J. Chem. Theory Comput. 11, 3584–3595. 10.1021/acs.jctc.5b00436 26300708 PMC4535365

[B27] Miao Y. McCammon J. A. (2016). Graded activation and free energy landscapes of a muscarinic G-protein–coupled receptor. Proc. Natl. Acad. Sci. U. S. A. 113, 12162–12167. 10.1073/pnas.1614538113 27791003 PMC5087018

[B28] Miao Y. McCammon J. A. (2018). Mechanism of the G-protein mimetic nanobody binding to a muscarinic G-protein-coupled receptor. Proc. Natl. Acad. Sci. U. S. A. 115, 3036–3041. 10.1073/pnas.1800756115 29507218 PMC5866610

[B29] Miao Y. Sinko W. Pierce L. Bucher D. Walker R. C. McCammon J. A. (2014). Improved reweighting of accelerated molecular dynamics simulations for free energy calculation. J. Chem. Theory Comput. 10, 2677–2689. 10.1021/ct500090q 25061441 PMC4095935

[B30] Michel M. C. Michel-Reher M. B. Hein P. (2020). A systematic review of inverse agonism at adrenoceptor subtypes. Cells 9, 1923. 10.3390/cells9091923 PMC7564766 32825009

[B31] Rasmussen S. G. F. Choi H.-J. Fung J. J. Pardon E. Casarosa P. Chae P. S. (2011a). Structure of a nanobody-stabilized active state of the β2 adrenoceptor. Nature 469, 175–180. 10.1038/nature09648 21228869 PMC3058308

[B32] Rasmussen S. G. F. DeVree B. T. Zou Y. Kruse A. C. Chung K. Y. Kobilka T. S. (2011b). Crystal structure of the β2 adrenergic receptor–Gs protein complex. Nature 477, 549–555. 10.1038/nature10361 21772288 PMC3184188

[B33] Roe D. R. Cheatham T. E. (2013). PTRAJ and CPPTRAJ: Software for processing and analysis of molecular dynamics trajectory data. J. Chem. Theory Comput. 9, 3084–3095. 10.1021/ct400341p 26583988

[B34] Ryckaert J.-P. Ciccotti G. Berendsen H. J. C. (1977). Numerical integration of the cartesian equations of motion of a system with constraints: Molecular dynamics of n-alkanes. J. Comput. Phys. 23, 327–341. 10.1016/0021-9991(77)90098-5

[B35] Salomon-Ferrer R. Case D. A. Walker R. C. (2013a). An overview of the Amber biomolecular simulation package. WIREs. Comput. Mol. Sci. 3, 198–210. 10.1002/wcms.1121

[B36] Salomon-Ferrer R. Götz A. W. Poole D. Le Grand S. Walker R. C. (2013b). Routine microsecond molecular dynamics simulations with AMBER on GPUs. 2. Explicit solvent particle mesh Ewald. J. Chem. Theory Comput. 9, 3878–3888. 10.1021/ct400314y 26592383

[B37] Stanek M. Picard L.-P. Schmidt M. F. Kaindl J. M. Hübner H. Bouvier M. (2019). Hybridization of β-adrenergic agonists and antagonists confers G protein bias. J. Med. Chem. 62, 5111–5131. 10.1021/acs.jmedchem.9b00349 31042379

[B38] Staus D. P. Strachan R. T. Manglik A. Pani B. Kahsai A. W. Kim T. H. (2016). Allosteric nanobodies reveal the dynamic range and diverse mechanisms of G-protein-coupled receptor activation. Nature 535, 448–452. 10.1038/nature18636 27409812 PMC4961583

[B39] Sun H. Li Y. Shen M. Tian S. Xu L. Pan P. (2014). Assessing the performance of MM/PBSA and MM/GBSA methods. 5. Improved docking performance using high solute dielectric constant MM/GBSA and MM/PBSA rescoring. Phys. Chem. Chem. Phys. 16, 22035–22045. 10.1039/C4CP03179B 25205360

[B40] Sun Z. Gong Z. Xia F. He X. (2021a). Ion dynamics and selectivity of Nav channels from molecular dynamics simulation. Chem. Phys. 548, 111245. 10.1016/j.chemphys.2021.111245

[B41] Sun Z. Huai Z. He Q. Liu Z. (2021b). A general picture of cucurbit[8]uril host–guest binding. J. Chem. Inf. Model. 61, 6107–6134. 10.1021/acs.jcim.1c01208 34818004

[B42] Tian C. Kasavajhala K. Belfon K. A. A. Raguette L. Huang H. Migues A. N. (2020). ff19SB: Amino-Acid-Specific protein backbone parameters trained against quantum mechanics energy surfaces in solution. J. Chem. Theory Comput. 16, 528–552. 10.1021/acs.jctc.9b00591 31714766 PMC13071887

[B43] Trincavelli M. L. Daniele S. Martini C. (2010). Adenosine receptors: What we know and what we are learning. Curr. Top. Med. Chem. 10, 860–877. 10.2174/156802610791268756 20370662

[B44] Wacker D. Fenalti G. Brown M. A. Katritch V. Abagyan R. Cherezov V. (2010). Conserved binding mode of human beta2 adrenergic receptor inverse agonists and antagonist revealed by X-ray crystallography. J. Am. Chem. Soc. 132, 11443–11445. 10.1021/ja105108q 20669948 PMC2923663

[B45] Wang J. Arantes P. R. Bhattarai A. Hsu R. V. Pawnikar S. Huang Y.-m. M. (2021). Gaussian accelerated molecular dynamics (GaMD): Principles and applications. Wiley Interdiscip. Rev. Comput. Mol. Sci. 11, e1521. 10.1002/wcms.1521 34899998 PMC8658739

[B46] Wang J. Miao Y. (2019). Mechanistic insights into specific G protein interactions with adenosine receptors. J. Phys. Chem. B 123, 6462–6473. 10.1021/acs.jpcb.9b04867 31283874 PMC7026936

[B47] Wang J. Miao Y. (2020). Peptide Gaussian accelerated molecular dynamics (Pep-GaMD): Enhanced sampling and free energy and kinetics calculations of peptide binding. J. Chem. Phys. 153, 154109. 10.1063/5.0021399 33092378 PMC7575327

[B48] Wang J. Wang W. Kollman P. A. Case D. A. (2006). Automatic atom type and bond type perception in molecular mechanical calculations. J. Mol. Graph. Model. 25, 247–260. 10.1016/j.jmgm.2005.12.005 16458552

[B49] Wang J. Wolf R. M. Caldwell J. W. Kollman P. A. Case D. A. (2004). Development and testing of a general amber force field. J. Comput. Chem. 25, 1157–1174. 10.1002/jcc.20035 15116359

[B50] Wu E. L. Cheng X. Jo S. Rui H. Song K. C. Dávila-Contreras E. M. (2014). CHARMM-GUI Membrane Builder toward realistic biological membrane simulations. J. Comput. Chem. 35, 1997–2004. 10.1002/jcc.23702 25130509 PMC4165794

[B51] Xu X. Kaindl J. Clark M. J. Hübner H. Hirata K. Sunahara R. K. (2021). Binding pathway determines norepinephrine selectivity for the human β1AR over β2AR. Cell. Res. 31, 569–579. 10.1038/s41422-020-00424-2 33093660 PMC8089101

[B52] Xue W. Wang P. Tu G. Yang F. Zheng G. Li X. (2018a). Computational identification of the binding mechanism of a triple reuptake inhibitor amitifadine for the treatment of major depressive disorder. Phys. Chem. Chem. Phys. 20, 6606–6616. 10.1039/C7CP07869B 29451287

[B53] Xue W. Yang F. Wang P. Zheng G. Chen Y. Yao X. (2018b). What contributes to serotonin–norepinephrine reuptake inhibitors’ dual-targeting mechanism? The key role of transmembrane domain 6 in human serotonin and norepinephrine transporters revealed by molecular dynamics simulation. ACS Chem. Neurosci. 9, 1128–1140. 10.1021/acschemneuro.7b00490 29300091

