# Supplementary material for: Exploring the deactivation mechanism of human β 2 adrenergic receptor by accelerated molecular dynamic simulations
Source: Front Mol Biosci. 2022 Aug 30;9:972463. doi: 10.3389/fmolb.2022.972463 (PMC9468641; doi:10.3389/fmolb.2022.972463)
Supplement: Supplementary file 1 [file DataSheet1.PDF]

Exploring the Deactivation Mechanism of Human β_2 Adrenergic Receptor by Accelerated Molecular Dynamic Simulations

Jianzhong Chen[1*], Jian Wang[1], Qingkai Zeng[1], Wei Wang[1], Haibo Sun[1] and Benzheng Wei[2*]

[1] School of Science, Shandong Jiaotong University, Jinan, 250357, China

[2] Center for Medical Artificial Intelligence, Shandong University of Traditional Chinese Medicine, Qingdao 266112, China

*Correspondence: Jianzhong Chen, chenjianzhong1970@163.com and jzchen@sdjtu.edu.cn

Benzheng Wei, wbz99@sina.com

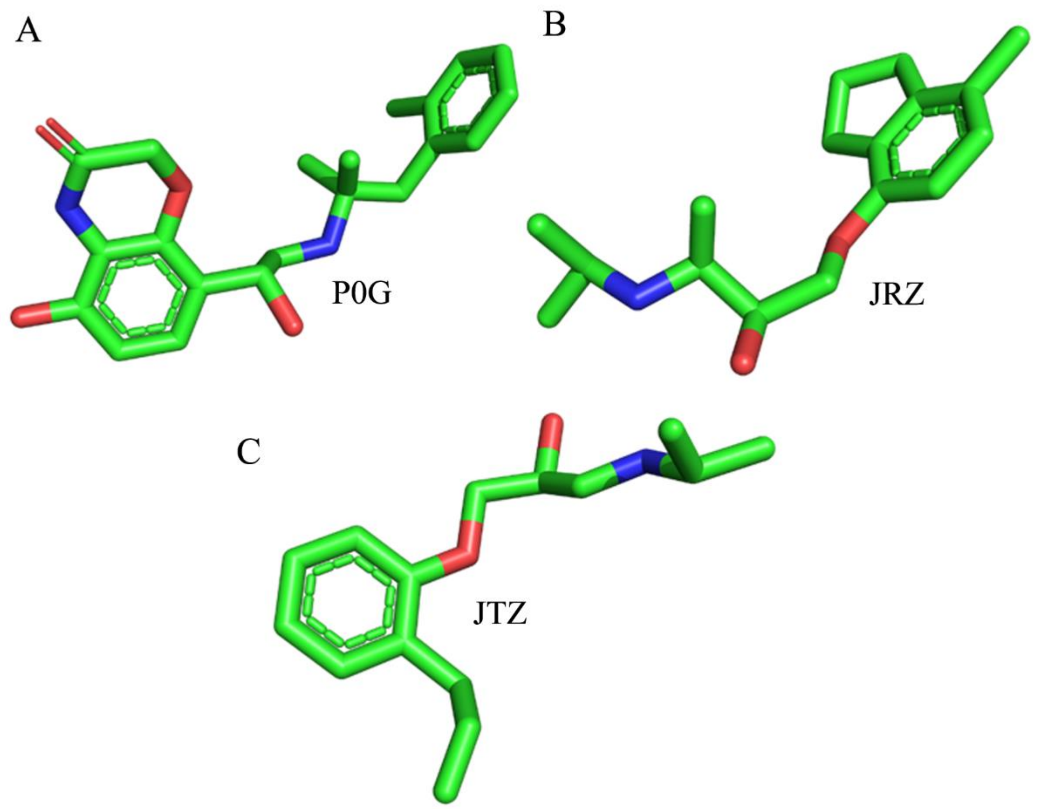

FIGURE S1 | Structures of ligands used in the current work: (A) agonist P0G, (B) inverse agonist JRZ and (C) antagonist JTZ.

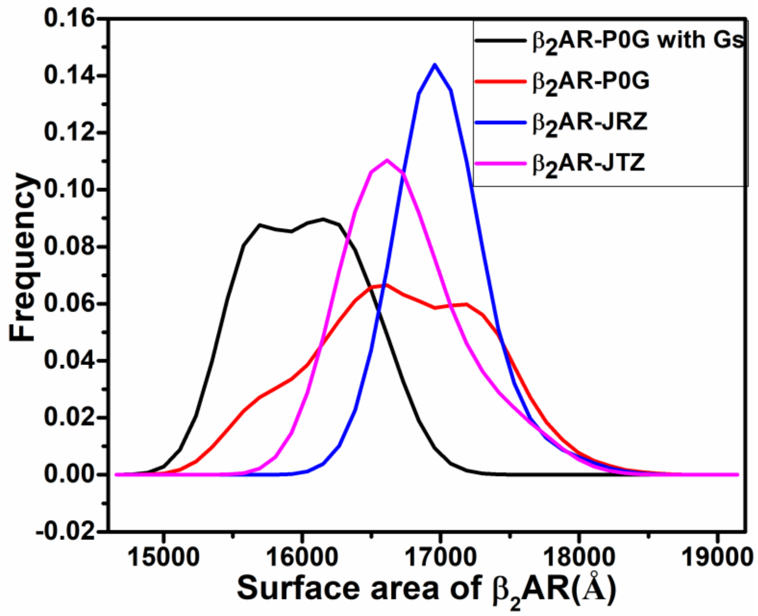

FIGURE S2 | Frequency distributions of molecular surface areas for the P0G-bound β_2AR with binding of the Gs proteins and the P0G-, JRZ- and JTZ-bound β_2AR without binding the Gs proteins.

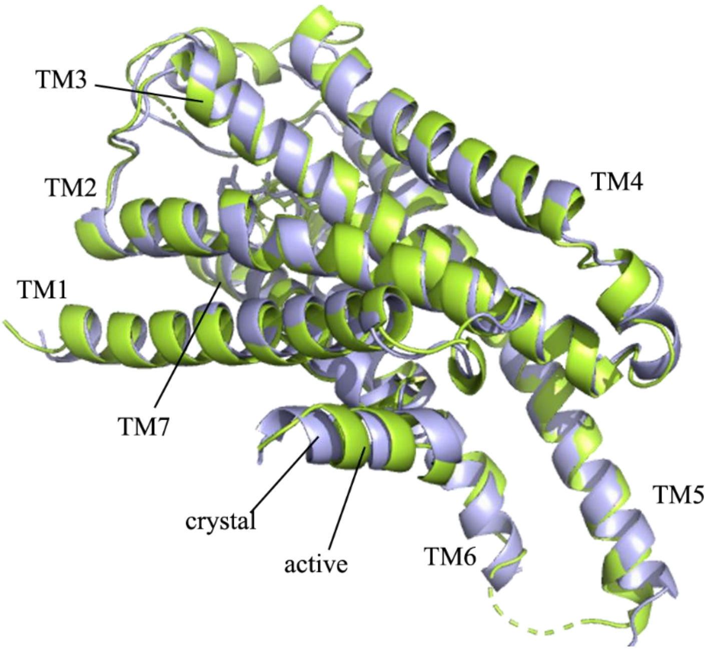

FIGURE S3 | Structural superimposition between the crystal structure of the P0G-bound β_2AR with the Gs protein and the active structure of the P0G-bound β_2AR with the Gs protein taken from GaMD simulations.

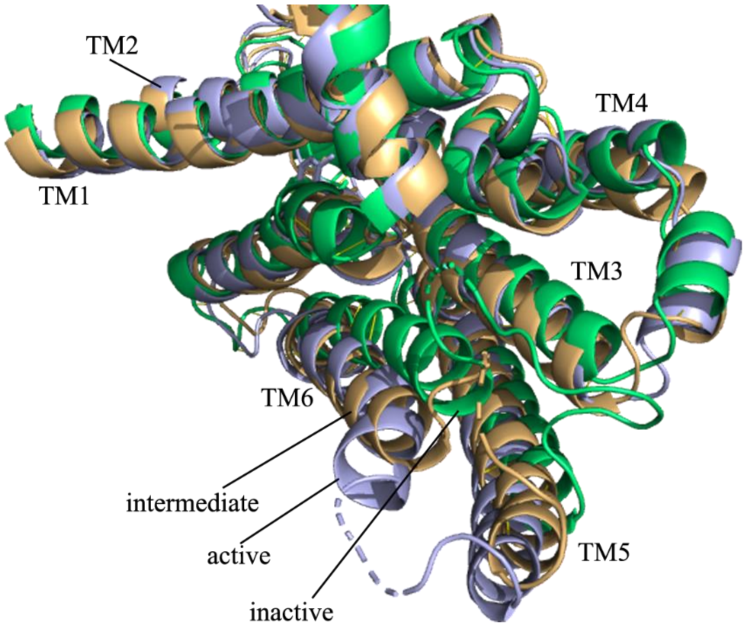

FIGURE S4 | Structural alignments of the P0G-bound active, intermediate and inactive β_2AR without the Gs protein.

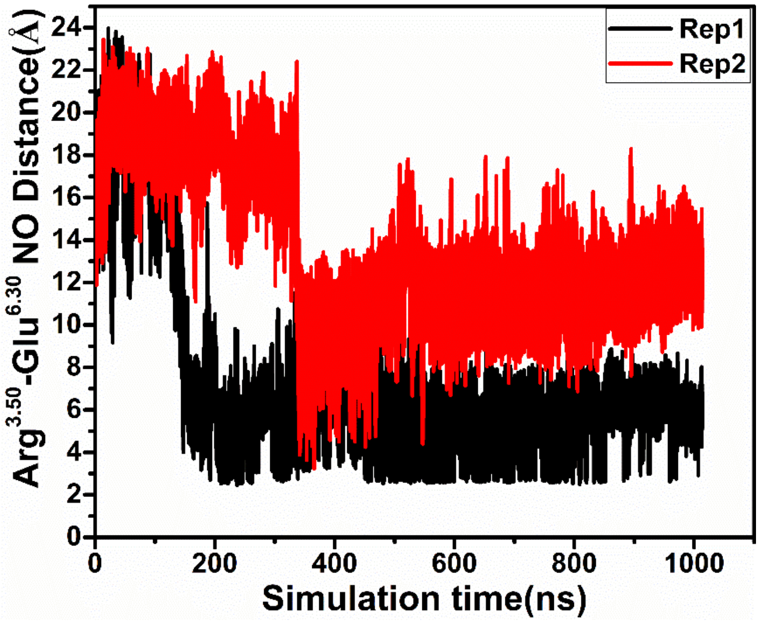

FIGURE S5 | Time evolution of the distance of the nitrogen atom (N) in $Arg^{3.50}$ away from the oxygen atom (O) in $Glu^{6.30}$ from the P0G-bound β_2AR without binding of the Gs proteins.

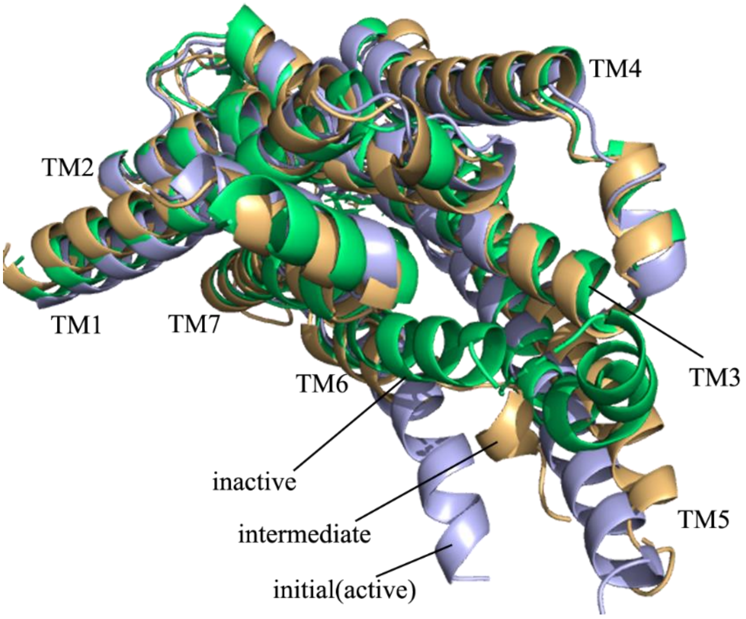

FIGURE S6 | Structural superimposition of the JRZ-bound active, intermediate and inactive β_2AR without binding of the Gs protein.

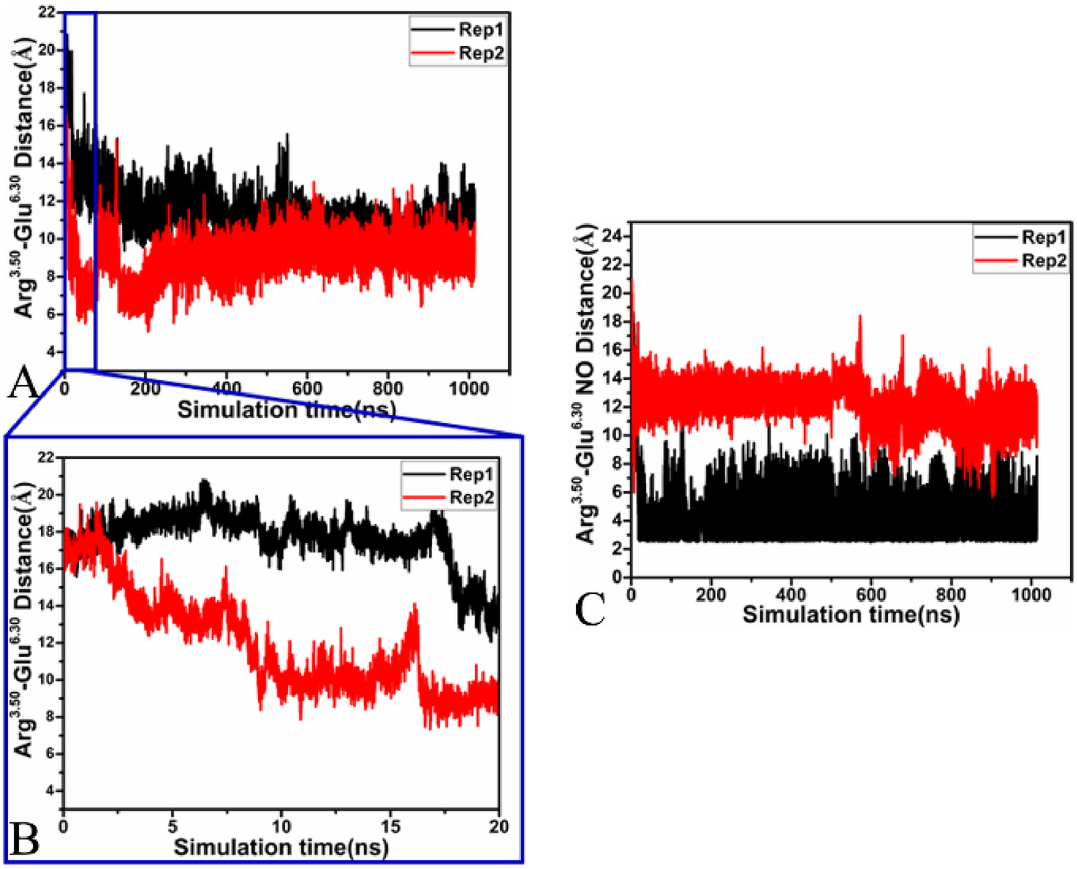

FIGURE S7 | Distance analysis relating with the simulated system of the JRZ-bound β_2AR without the Gs proteins: (A) evolution of the distance between the C_α atoms of $Arg^{3.50}$ and $Glu^{6.30}$ as simulation time in the JRZ-bound active β_2AR without the Gs proteins, (B) the changes in the distance between the C_α atoms of $Arg^{3.50}$ and $Glu^{6.30}$ from the first 20 ns of the equilibrium phase and (C) Time evolution of the distance of the nitrogen atom (N) in $Arg^{3.50}$ away from the oxygen atom (O) in $Glu^{6.30}$.

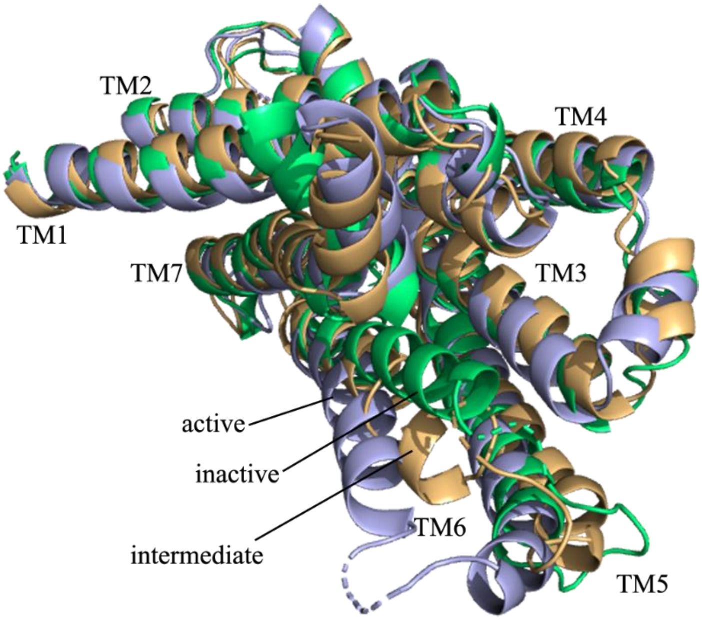

FIGURE S8 | Structural superimposition of the JTZ-bound active, intermediate and inactive β_2AR without binding of the Gs protein.

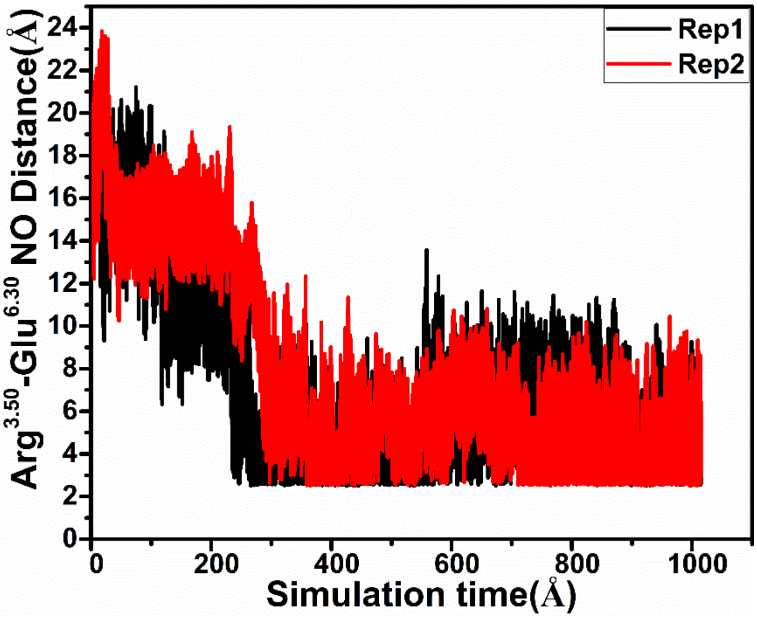

FIGURE S9 | Time evolution of the distance of the nitrogen atom (N) in $Arg^{3.50}$ away from the oxygen atom (O) in $Glu^{6.30}$ from the JTZ-bound β_2AR without binding of the Gs protein.

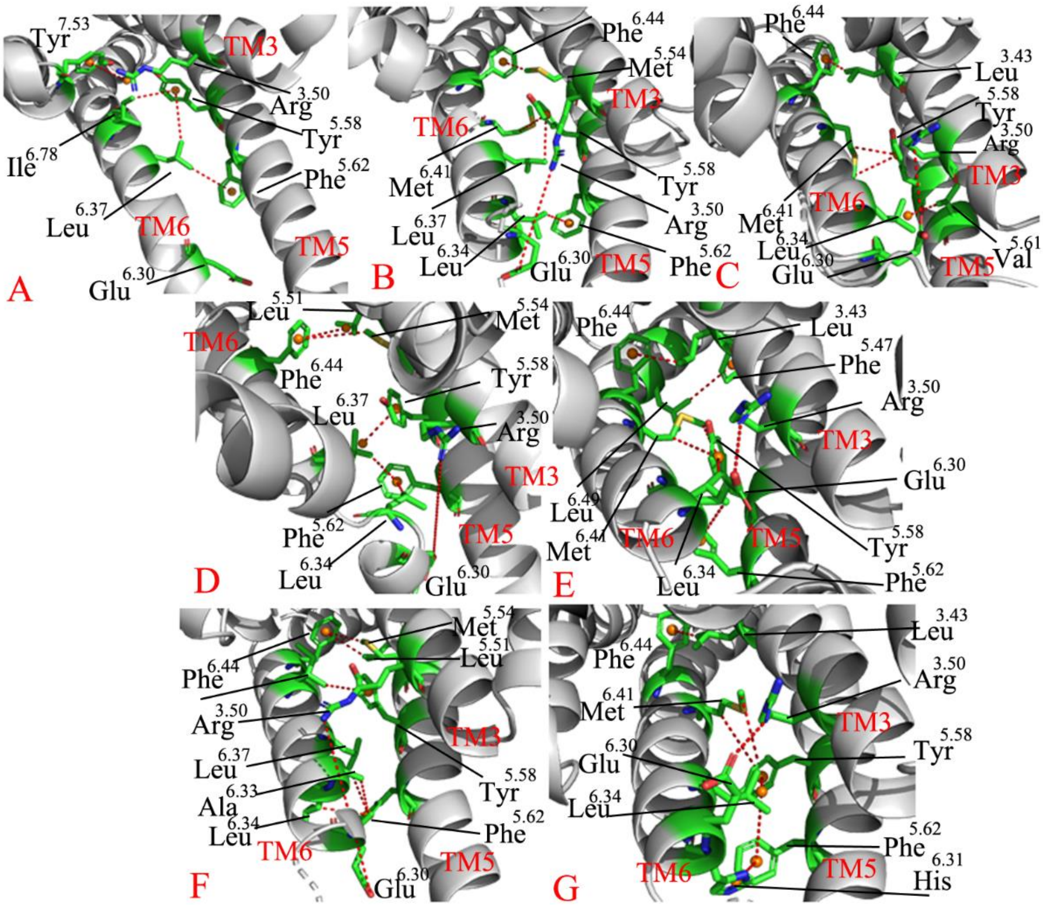

FIGURE S10 | Interactions of key residues from helixes TM3, TM5 and TM6 in the active, intermediate and inactive states of the β_2AR: (A) the active β_2AR, (B) and (C) corresponding to the P0G-bound intermediate and inactive states of the β_2AR, respectively, (D) and (E) representing the JRZ-bound intermediate and inactive states of the β_2AR, separately, and (F) and (G) indicating the JTZ-bound intermediate and inactive states of the β_2AR, individually.

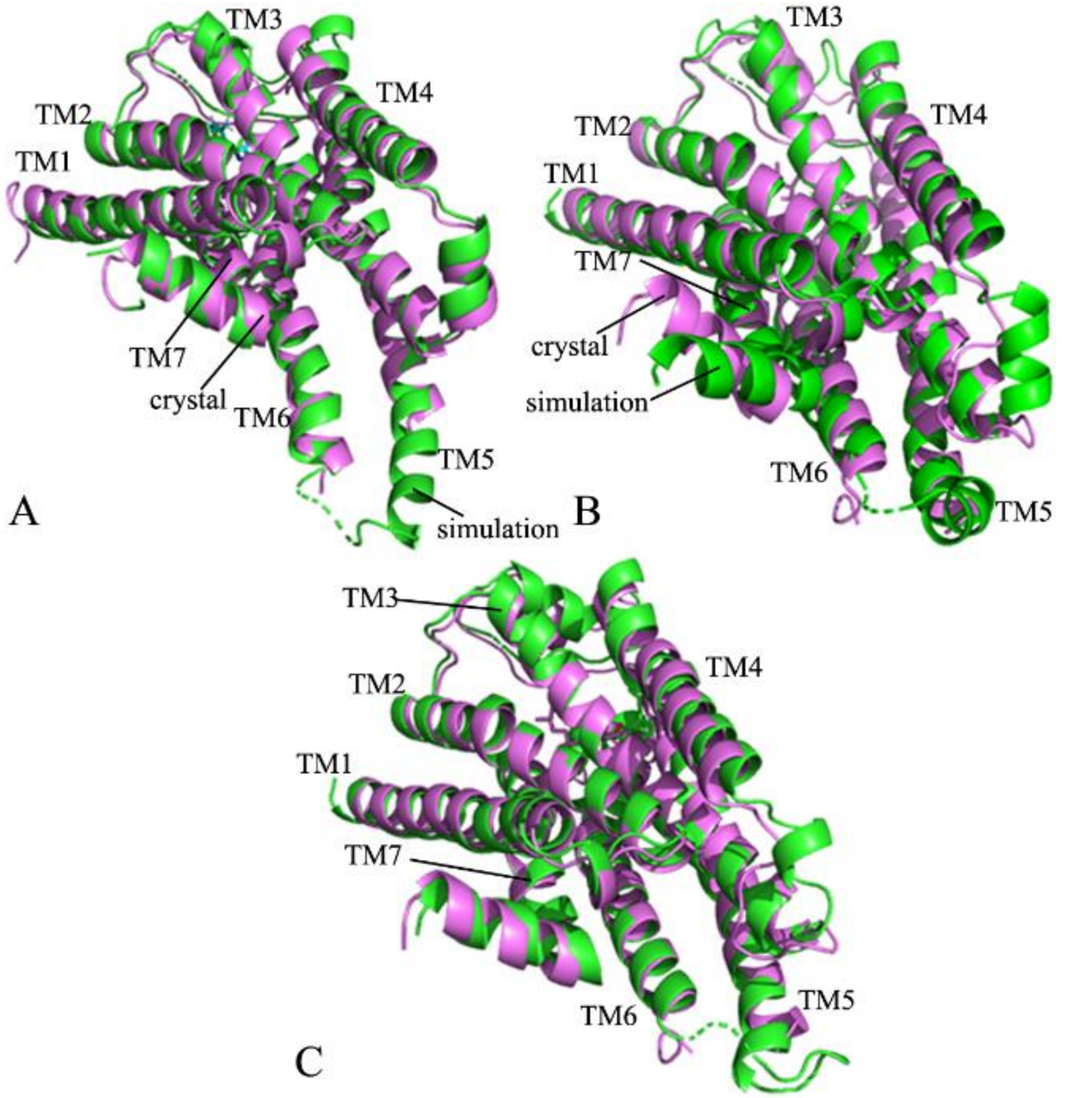

Figure S11. (A) Structural superimposition between the active state of the P0G-bound β_2AR without the Gs protein captured by GaMD simulations and its crystal structure 3P0G, (B) Structural alignment between the inactive state of the JRZ-bound β_2AR without the Gs protein captured by GaMD simulations and its crystal structure 3NY8 and (C) Structural superimposition of the inactive state of the JTZ-bound β_2AR without the Gs protein captured by GaMD simulations and its crystal structure 3NYA.
